# The Nutriment of Beer

**Published:** 1868-10

**Authors:** 


THE NUTRIMENT OF BEER.—People who drink their ale and beer are very fond of telling how much nutriment they derive from them. Because they are manufactured from grain, many have the idea that the concentrated virtues of the grain are in the drinks. This is an entire fallacy. Prof. Liebig, one of the most eminent chemists in the world, assures us that 1,460 qts. of the best Bavarian beer contain exactly the nourishment of a two-and-a-half pound loaf of bread. This beer is very similar to the famous English Allsopp's and our more popular American beer. The fact is, the nutritious portion of the grain is rotted out before the beer can be made; and if the fermentation of the beer has been complete, Prof. Lyon Playfair declares that no nourishment whatever remains in the fermented liquor; and, as the English Alliance News says, "No chemist now disputes this assertion; for, except to flavor and amount of alcohol, the chemical composition of all kinds of beer is alike and brewers must laugh to hear doctors advising porter as more nourishing than beer, when porter is nothing but beer colored by burnt malt; and often when beer goes wrong in the making and is unsaleable beer, it is converted into fine porter, the mere color covering many defects."—*Journal of Chemistry.*—*Richmond & Louisville Medical Journal.*

